# The Promise of Selective PARP1 Inhibitors in Cancer Therapy

**DOI:** 10.1021/acsmedchemlett.5c00561

**Published:** 2025-10-08

**Authors:** Xin Zhou, Jimmy S. Patel, Taoqian Zhao, Steven H. Liang

**Affiliations:** Department of Radiology and Imaging Sciences, 1371 Emory University, 1364 Clifton Road, Atlanta, Georgia 30322, United States

**Keywords:** Poly(ADP-ribose) polymerase 1 (PARP1), Poly(ADP-ribose) polymerase inhibitor (PARPi), DNA damage response; homologous recombination deficiency (HRD), anticancer, structure−activity relationship (SAR)

## Abstract

Poly­(ADP-ribose) polymerase 1 (PARP1) is a critical enzyme involved in the DNA damage response. The therapeutic inhibition of PARP1 has emerged as a promising strategy, particularly in cancers with homologous recombination deficiencies (HRDs). A recent report disclosed the discovery of (*S*)-G9 as a selective PARP1 inhibitor for clinical development.

## Graphical abstract

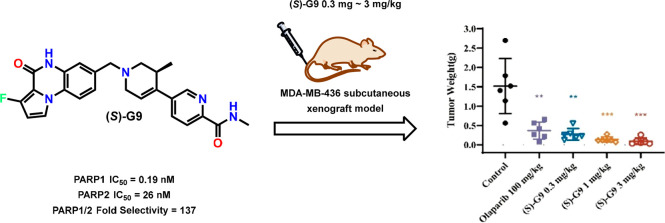

Poly­(ADP-ribose) polymerase 1 (PARP1) is a critical nuclear enzyme instrumental in maintaining genomic integrity and plays a significant role in detection and repair of single-strand breaks (SSBs) via base excision repair (BER) pathway.^1,2^ The activation of PARP1 triggers poly­(ADP-ribosyl)­ation (PARylation) of itself and other target proteins (e.g., chromatin proteins), facilitating the recruitment of DNA repair machinery and modulating chromatin structure to maintain DNA stability.^3,4^ The therapeutic inhibition of PARP1 induces an accumulation of DNA lesions that lead to synthetic lethality in cancers with homologous recombination (HR) deficiencies (e.g., those harboring BRCA1/2 mutations).^5–7^ This principle underpins the clinical success of PARP inhibitors (PARPi) in oncology^8,9^ (Figure 1).

**Figure 1:**
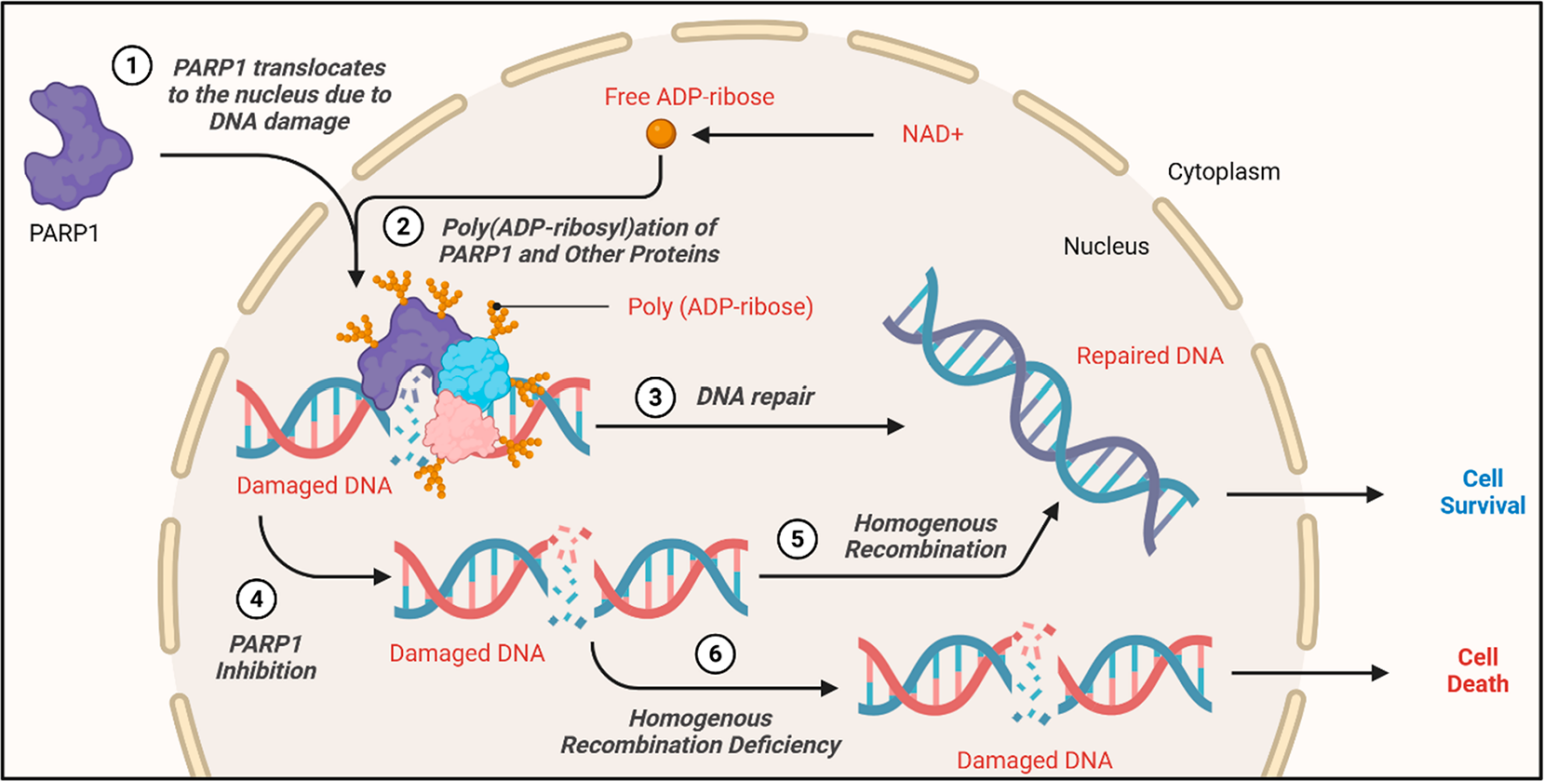
Mechanism and function of PARP1 in DNA repair. (1). PARP1 enzyme translocate to the nucleus when DNA damage is detected. (2). PARP1 enzyme binds to damaged DNA and catalyzes the poly­(ADP-ribosyl)­lation of itself and other related proteins. (3). DNA repaired via PARP1 mediated BER pathway. (4). PARP1 inhibition. (5). Cells rely on homologous recombination pathway for DNA repair when PARP1 enzyme is inhibited. (6). The damaged DNA will lead to cell death when the cells are PARP1 inhibited and homologous recombination deficient. This figure was created using BioRender (BioRender.com) under a publication license.

However, first-generation PARPi (e.g., Olaparib, Niraparib) are nonselective, potently inhibiting both PARP1 and the highly homologous PARP2 isozyme.^10–13^ This lack of selectivity is now understood to be a primary contributor to their dose-limiting toxicities, particularly myelosuppression and anemia.^14^ While genetic knockout studies confirm that single PARP1 or PARP2 deficiency is viable in model organisms, dual PARP1/PARP2 knockout is embryonically lethal.^15^ Critically, PARP2-specific deficiency has been demonstrated to impair erythrocyte progenitor cell differentiation, shorten red blood cell lifespan, and result in chronic anemia, highlighting its distinct and essential role in hematopoiesis.^16^ As the synthetic lethal interaction with BRCA deficiency is primarily mediated through PARP1 inhibition, the concomitant inhibition of PARP2 is not required for efficacy and instead contributes to clinical toxicity.^17^ Consequently, a compelling strategic shift in the field is toward developing highly selective PARP1 inhibitors to maximize the therapeutic window.

This rationale has driven the development of next-generation, PARP1-selective compounds. AZD5305, a leading candidate from AstraZeneca’s portfolio, exemplifies this approach.^19,20^ Co-crystal structures reveal that inhibitors like AZD5305 anchor into the PARP1 NAD^+^ binding site through a quinazolinone scaffold. The amide bond on the quinazolinone core forms three critical hydrogen bonds with backbone atoms of Gly863 and Ser904. A second amide moiety engages in a hydrogen bond with the backbone of Ile879 (2.2 Å). The planar quinazolinone system engages in stabilizing π-stacking interactions with Tyr907. An ethyl substituent projects into a hydrophobic subpocket defined by residues Tyr907, Ala898, and Glu988. Analysis of the AZD5305-PARP1 cocrystal structure reveals that the ethyl group does not fully occupy the available hydrophobic space within this subpocket. This presents a clear opportunity for molecular optimization. (Figure 2) Recent research reported by Guo and co-workers focuses on the systematic modification of the quinazolinone scaffold’s terminus to incorporate ring-constrained structures (Figure 1, lead molecule G9).^18^ The introduction of a larger, rigid hydrophobic moiety at the ethyl position is hypothesized to significantly enhance both binding affinity and selectivity. This improvement is anticipated through two mechanisms: enhanced shape complementarity with the target binding pocket, optimizing van der Waals interactions, and a more favorable entropy-driven binding process. The latter is achieved by displacing water molecules from the hydrophobic region of the active site, thereby reducing the entropic penalty associated with ligand binding. This rational design strategy aims to achieve compounds with robust antitumor efficacy and an improved therapeutic safety profile.

**Figure 2:**
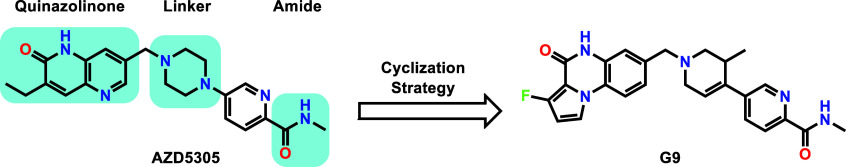
Design of novel PARP1 inhibitors based on AZD5305. Structure-based design of the PARP1 inhibitors. The figure was adapted from ref 18. Copyright 2025 American Chemical Society.

Inspired by the hypothesis, the preliminary work confirmed that the cyclized quinazolinone core structure provided the most effective foundation for inhibitor development and offered superior potency and properties compared to its acyclic predecessors. Subsequent efforts were directed toward the linker region. Systematic exploration led to the identification of a cyclopropyl-substituted piperazine as the optimal linker moiety. This group was found to confer a conformational advantage that enhanced binding affinity and potency within the PARP enzyme active site. With the core and linker regions firmly established, the campaign progressed to a comprehensive exploration of the substituent space. A systematic structure–activity relationship (SAR) study was conducted by synthesizing and testing analogues with diverse modifications at the R_4_, R_5_, R_6_, R_7_, and R_8_ positions.^21^ Each analogue was rigorously evaluated using standardized PARP1 and PARP2 enzymatic assays to determine *in vitro* inhibitory potency and crucially selectivity between the two PARP isoforms (Scheme 1). This extensive SAR investigation culminated in the identification of compound G9. This compound emerged as the lead candidate, distinguished by its exceptional potency against PARP1 and a favorable selectivity profile over PARP2. This combination of high potency and selectivity positions G9 as a promising candidate for further preclinical development.

**Scheme 1:**
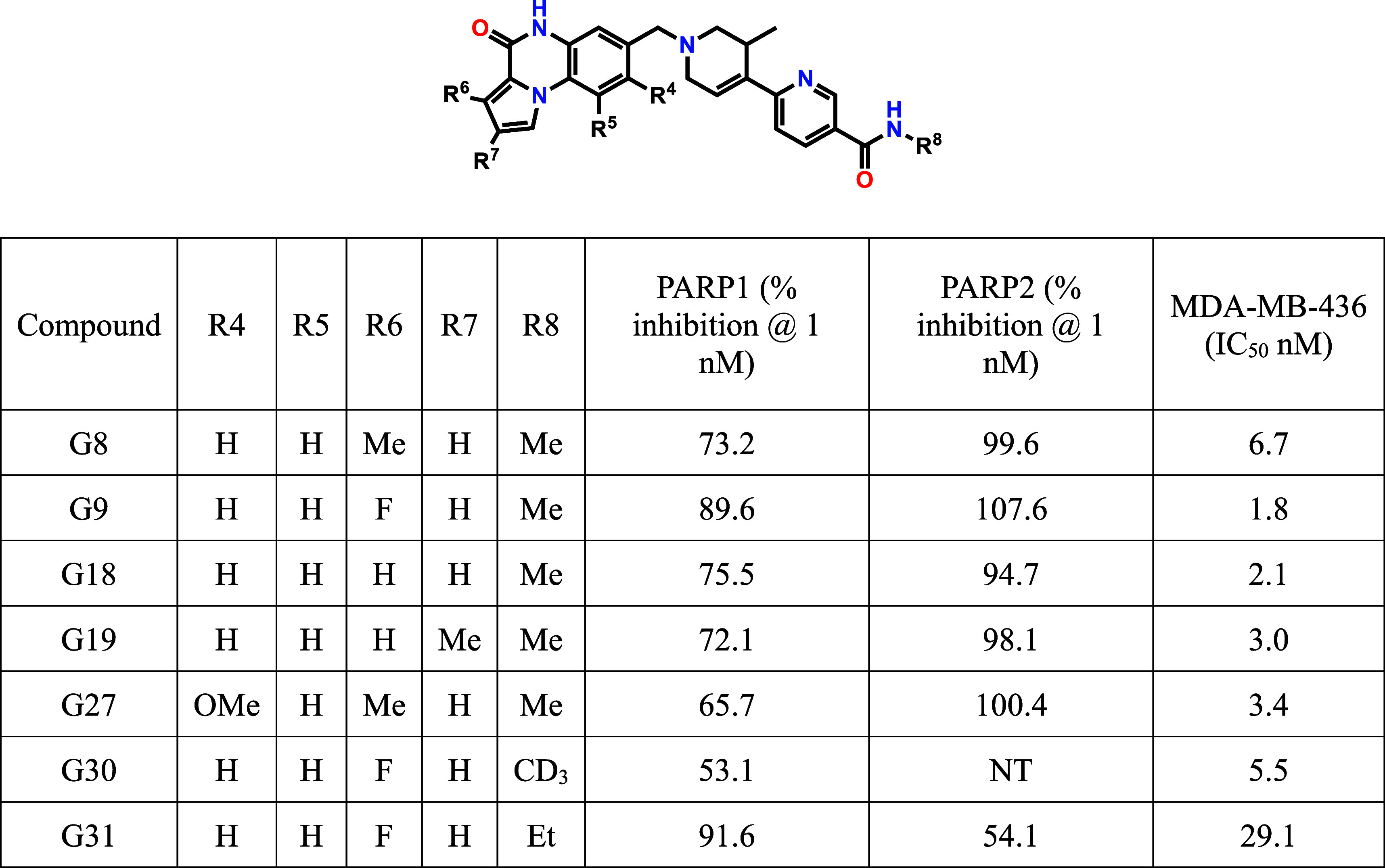
All Compounds Were Assayed at Least Twice, and the Inhibitory Values Were Averaged.^a^

a All IC_50_ values are the average of three replicates. NT, not tested. The Scheme Was Adapted from Ref 18. Copyright 2025 American Chemical Society.

The presence of a chiral center in the lead compound G9 prompted the synthesis and individual evaluation of both enantiomers. Subsequent profiling identified the (*S*)-G9 is a highly potent and selective nanomolar inhibitor of PARP1 compared to (*R*)-G9. In biochemical assays, (*S*)-G9 demonstrated superior potency and exceptional selectivity in PARP1 enzymatic activity with an IC_50_ of 0.19 nM, a 4-fold improvement in potency over Olaparib (IC_50_ = 0.8 nM). (*S*)-G9 exhibited minimal activity against PARP2 (IC_50_ = 26.0 nM), 137-fold selectivity for PARP1 over PARP2, in contrast to Olaparib, which demonstrates equal potency against PARP2 and PARP1 (PARP1 IC_50_ = 0.6 nM, PARP2 IC_50_ = 0.5 nM; Table 1). The selectivity profile was further confirmed by a lack of off-target activity across a panel of 104 kinases. (*S*)-G9 inhibited PARylation and induced selective cytotoxicity in BRCA mutant cell lines. In a cellular PARP1 knockout (KO) A549 model, both (S)-G9 and Olaparib lost potency (IC_50_ > 1000 nM), confirming that their cytotoxic effects are PARP1-dependent. However, in a PARP2 KO A549 model, (*S*)-G9 maintained potent activity (IC_50_ = 3.5 nM), while the efficacy of Olaparib was drastically reduced (IC_50_ = 2301 nM). This key experiment provides direct cellular evidence for superior reliance on PARP1 of (*S*)-G9 inhibition for its antitumor effect, distinguishing it from the dual PARP1/PARP2 inhibitory profile of Olaparib. In addition, (*S*)-G9 proved to be a best-in-class inducer of PARP1-DNA complexes (trapping), exhibiting >900-fold selectivity for PARP1 over PARP2 trapping. This potent trapping activity translated to a robust mechanistic response, characterized by increased DNA damage (as measured by γH2AX formation, which is a marker for double-stranded breaks) and subsequent G2/M cell cycle arrest. Supported by promising best-in-class pharmacokinetic properties of high oral exposure, a long elimination half-life (∼24–27 h in mice), and complete oral bioavailability, which makes (S)-G9 a potential clinical candidate amenable to once-daily dosing.

**Table 1:** Selectivity Profile of (*S*)-G9 and Olaparib across 2 PARP Isoforms^a^

Biochemical assay	Olaparib	(*S*)-G9
PARP1 IC_50_ (nM)	0.8	0.19
PARP2 IC_50_ (nM)	0.5	26
PARP1/2 fold Selectivity	0.6	137

a The Table Was Adapted from Ref 18. Copyright 2025 American Chemical Society.

Following promising *in vitro* results, the therapeutic potential of (*S*)-G9 was evaluated *in vivo*. The compound demonstrated exceptional efficacy as a monotherapy in a BRCA1 mutant MDA-MB-436 xenograft model, inducing robust and dose-dependent tumor regression at a low dose of 0.3 mg/kg. (Figure 3) This effect significantly surpassed that of Olaparib dosed at 100 mg/kg. Furthermore, (*S*)-G9 exhibited strong synergistic antitumor activity when combined with liposomal irinotecan in an HCT116 xenograft model, which indicated its potential for combination chemotherapy regimens. Critical to its clinical translation, acute and subacute toxicity studies in mice revealed no unacceptable adverse effects or histopathological changes in major organs, indicating a wide therapeutic window. This favorable safety profile is a key advantage over less selective PARP inhibitors. The development of (*S*)-G9 is situated within the broader pursuit of next-generation PARP1-selective inhibitors, a class exemplified by clinical candidate AZD5305, which aims to improve upon the safety limitations of first-generation dual PARP1/2 inhibitors like Olaparib.

**Figure 3:**
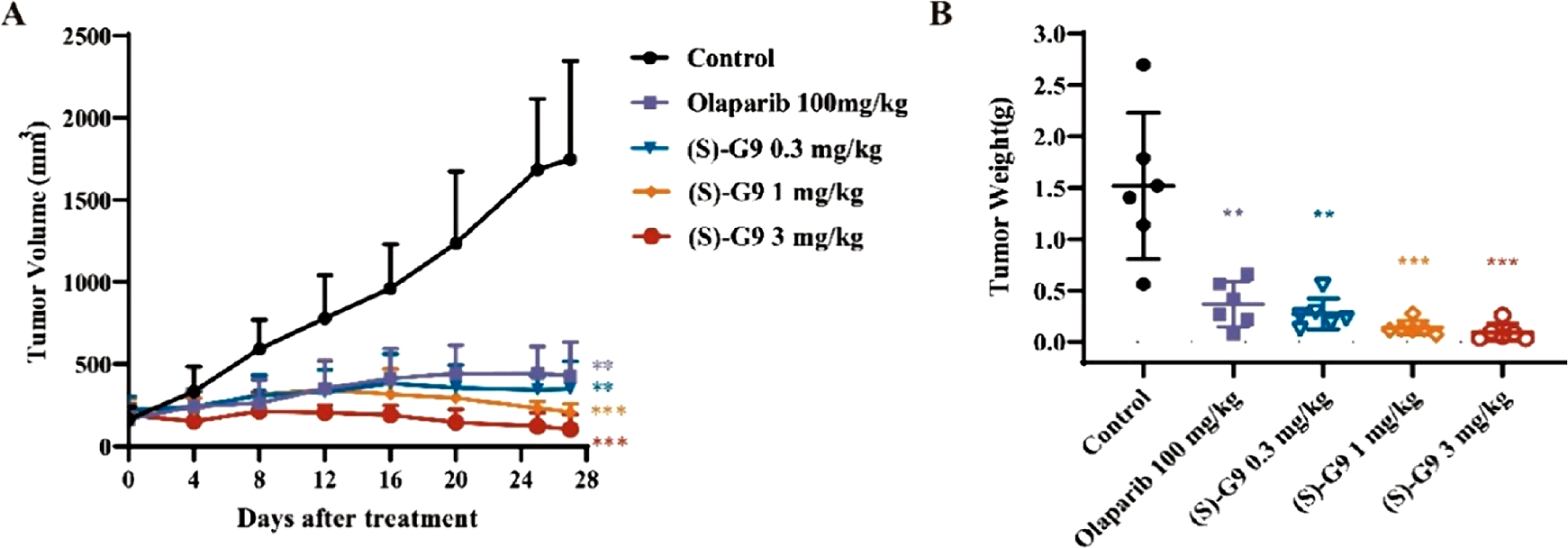
*In vivo* antitumor efficacy of (*S*)-G9 in an MDA-MB-436 subcutaneous xenograft model. (A) Tumor volume curve of xenograft in nude mice during treatment. (B) Tumor weight at the end of treatment. The figure was adapted from ref 18. Copyright 2025 American Chemical Society.

In conclusion, (*S*)-G9 emerges as a highly selective PARP1 inhibitor. Its superior PARP1 selectivity, potent *in vivo* efficacy at minimal doses, synergistic potential in combination therapy, and clean preclinical safety profile position it as a promising clinical candidate. The cyclization strategy employed herein provides a validated and innovative design paradigm for developing safer and more efficient PARP1-targeted therapies.

## Outlook

The development of (*S*)-G9 represents a significant advancement in the field of PARP inhibition and addresses a critical limitation of first-generation PARP inhibitors. The lack of selectivity between PARP1 and PARP2 makes first-generation PARP inhibitors constrained by dose-limiting hematological toxicities.^22^ The exceptional selectivity profile, potent enzymatic and cellular activity, favorable pharmacokinetics, and robust *in vivo* efficacy of (*S*)-G9 positions it as a strong candidate for clinical development. (*S*)-G9 demonstrates superior oral bioavailability (100% in mice, 89% in rats) and a long half-life, supporting the potential for once-daily dosing in humans. Its high potency at low doses (0.3–3 mg/kg), compared to Olaparib (100 mg/kg), suggests a wider therapeutic window and a reduced risk of off-target effects. Moreover, its clean kinase selectivity profile and minimal PARP2 trapping mitigate the likelihood of hematological and other dose-limiting toxicities, which is a common drawback of dual PARP1/2 inhibitors.^23^

Future studies could explore combinations of (*S*)-G9 with other DNA-damaging agents, immunotherapies, or targeted therapies in both BRCA mutant and HRD cancers. Future analog development may focus on enhancing CNS penetration, solubility, and metabolic stability based on the established SAR. With PARP1-selective compounds such as AZD5305 ^20^ and AZD9574 ^24^ already in clinical trials, long-term toxicology studies and the monitoring of resistance mechanisms (e.g., PARP1 mutations, restoration of HR function) will be essential to fully define the clinical utility of (*S*)-G9. As the field advances toward precision oncology, (*S*)-G9 and its successors hold great promise for improving outcomes in patients with DNA repair-deficient cancers, both as monotherapies and in combination regimens. Ongoing efforts should prioritize clinical translation of (*S*)-G9, expansion of its therapeutic applications, and refinement of next-generation PARP1-selective inhibitor design.

## Abbreviations

PARP: poly­(ADP-ribose) polymerase
PARPi: poly­(ADP-ribose) polymerase inhibitor
BRCA: breast cancer
SAR: structure–activity relationship
SSBs: single-strand breaks
BER: base excision repair
HR: homologous recombination
CNS: central nervous system; TGI: tumor growth inhibition.
